# Social prescribing for people with mental health needs living in disadvantaged communities: the Life Rooms model

**DOI:** 10.1186/s12913-019-4882-7

**Published:** 2020-01-06

**Authors:** Shaima M. Hassan, Clarissa Giebel, Esmaeil Khedmati Morasae, Clare Rotheram, Virginia Mathieson, Daniel Ward, Vicky Reynolds, Alan Price, Katie Bristow, Cecil Kullu

**Affiliations:** 1 Institute of Population Health Sciences, University of Liverpool, Liverpool, UK; 2 NIHR CLAHRC NWC, Liverpool, UK; 3 NIHR ARC NWC, Liverpool, UK; 4 Mersey Care NHS Foundation Trust, Liverpool, UK

**Keywords:** Mental health, Social prescribing, Disadvantaged communities, Socio-economic factors

## Abstract

**Background:**

People live socially complex lives and have different health care needs influenced by socio-economic factors such as deprivation, unemployment, and poor housing. Lack of access to community based social care results in people seeking social support from health care services. This study explores the Life Rooms as a social prescribing model addressing the social determinants of mental health by providing support and access to resources in a local community setting. With an aim to identify key elements that contribute toward enhancing the effectiveness of the Life Rooms social prescribing approach.

**Methods:**

Data were obtained through six semi-structured focus groups with mental health service users from two locations in the North West of the UK. Postcode data was collected to generate an Index for Multiple Deprivation (IMD) score, to understand their socio-economic background. Data were analysed using thematic analysis.

**Results:**

A total of 18 participants took part in the study. The majority of participants came from disadvantaged backgrounds; 14 participants measuring 3 and below in terms of overall IMD scores and 9 participants belonged to the poorest decile (IMD score = 1). Participants reported on different elements of the Life Rooms which they found as an effective approach to care. Four main themes emerged from the data: 1) social belonging: being able to just ‘be’ 2) resourceful and accessible; 3) social inclusion and connectedness; and 4) moving forward: self-development and independence.

**Conclusion:**

Findings support the need and benefit social prescribing to improve mental health wellbeing and reduce the burden of mental illness.

## Background

Mental health problems can affect anyone. Research shows that 1 in 6 adults in the UK experience a mental health problem in any given week [1]. Mental health problems such as depression and anxiety not only cause significant emotional distress and interrupt daily function for the individual, but also affect their families, community, and wider society [1].

Despite the ubiquitous nature of mental health problems, they disproportionately affect those from a poorer socio-economic background [2]. Poor mental health and mental illness are associated with socio-economic factors such as poverty, unemployment, low educational attainment, and poor housing, which are also linked to high rates of physical health problems [2]. Such factors are strongly linked to living socially complex lives and experiencing complex needs [3]. Every individual does not have a single need, but multiple inter-connected needs that span medical and social issues [3]. For example, an individual may have mental health problems, combined with experiences of social exclusion issues such as temporal/unsuitable accommodation or homelessness, substance misuse, street culture activities (such as street drinking), and institutional care (such as prison, local authority care, mental health hospitals) [3, 4]. The way people respond to such complexity (or multiple inter-linked issues) in their lives differ from one person to another. Therefore, people with complex needs require a new type of service as well as a new response from existing services in addressing social factors and reducing health inequalities [3, 5].

This requires a whole person approach which addresses the root causes of poor health and emphasises the need for integrated health and social care in the community [6]. This becomes more apparent in an individual’s care journey on transitioning from one care setting to another [6]. For example, not being able to access appropriate care and support within a community setting can result in an individual being in a medical care setting that is disorder and symptom orientated, rather than a social care setting that focuses on quality of life and social support [6, 7].

The approach to social prescribing (SP) is not new within the field of mental health. The ‘*Saving Lives: our healthier nation’* in 1999 promoted the better utilisation of community services [8], and further advocated by the Department of Health White Paper [9] and the NHS England [10]. More recently, the Secretary of state for Health advocated this holistic non-clinical approach through SP [7, 10, 11]. There are many models of SP, however, common to all of the models is an aim is to make better use of community initiatives to improve wellbeing [10, 12]. SP is commonly described as a mechanism that links primary care users with non-clinical support in the local community.

The principles of SP are highlighted in the NHS five year forward view [13, 14], which emphasises the focus on community engagement, prevention, patient-centred care and integrated services. This approach includes making use of the voluntary and community sector to offer a variety of practical information and activities to help address psychological problems and low levels of wellbeing [10, 12]. More recently, the NHS Long Term Plan specifically referenced SP in the context of diversifying the range of support on offer to NHS patients [15].

The wider literature demonstrates positive outcomes of SP, such as improvements in quality of life, emotional and overall mental wellbeing, and reducing social exclusion for disadvantaged groups [12, 16–19]. It also highlights that SP encompasses a variety of approaches, which is based on creating greater collaboration between healthcare settings and community-based health and social care settings [19]. The approaches vary from signposting to facilitate access of support without formal referrals, to a more comprehensive referral approach from primary care settings to a specific community-based programme. These approaches provide the potential to respond effectively to the impact of the social determinants of health [19].

Although SP is currently advocated across UK healthcare services, there is no specific guidance on what SP should entail [12]. However, different elements are highlighted that make up SP, such as providers having local knowledge and operating within a local remit, integrated care, holistic and individualised care to enhance overall wellbeing [12, 16–18].

In line with a SP approach, the aim of the Life Rooms is to address social determinants of mental health within two locations (Liverpool and Sefton) in the North West Coast (NWC) of the UK. The NWC is one of the most disadvantaged areas in the country, with consistently higher rates of premature mortality, illness, hospital admissions, mood and anxiety disorder [20]. Specifically, the NWC Household Health Survey (HHS) has shown how socio-economic issues are linked to variations in healthcare utilisation and suggests the need to address both socio-economic issues and structural issues, such as public transport and access to primary care [21, 22]. This study explored the experiences of secondary mental health care service users use of the Life Rooms. In particular, it explored how the support and access to resources provided by the Life Rooms addressed the social determinants of mental health. We hypothesise that knowledge from this study will provide further insight into the key elements that contribute toward enhancing the effectiveness of the Life Rooms social prescribing approach, which will further inform the implementation of additional Life Rooms hubs and other SP initiatives that operate in similar settings.

## Method

### The Life Rooms model

The Life Rooms is a service run by Mersey Care NHS Foundation Trust with a key co-production principle that emphasises a side by side with service users, carers, partner organisations and staff. In this way, the development of the service is informed by an ethos of lived experience and aims to meet the social needs of the communities in which they are located.

Upon accessing the Life Rooms, visitors are welcomed by staff and given the opportunity to explore support options through an informal and collaborative discussion with staff. This includes staff who have received specialist training in mental health, peer tutors (experienced in different areas such as anxiety, OCD, mindfulness, etc.), experienced mental health facilitators, chaplain and/or volunteers who have experience of mental distress. Support accessed is grounded in the choice of the individual, but typically takes the form of learning opportunities or social support.

The Life Rooms model places significant focus on recovery, and the recovery college is a key component of the model. The Life Rooms provides learning opportunities, delivering free courses covering a range of topics such as wellbeing, understanding and management of mental distress, social factors related to mental distress and other social and creative offerings. Another key part of the Life Rooms model is the pathways advice service, this delivers social prescribing support through a one-to-one daily drop in with a pathways advisor experienced in housing, debt and employment matters. Alongside a pathways advisor and in line with the Life Rooms co-production principle, someone using the Life Rooms can co-create their own social prescription; including things like housing, debt, employment or wellbeing support. An employment and enterprise volunteering support is also available at the Life Rooms, with many volunteering roles on offer within the service.

Additionally, the Life Rooms model provide safe spaces and welcoming environments, with access to community resources such as library services, computers and café facilities. The Life Rooms are open to all without any formal requirements for access. People attending the Life Rooms include Mersey Care service users and carers, people under primary care or receiving support from public or third sector organisations, as well as the general public. Whilst some individuals that access the Life Rooms may have received a referral from a clinician, others access the Life Rooms through self-referral.

### Study design

This study comprised of qualitative semi-structured focus groups to explore service users’ experiences of the Life Rooms and its impact on their mental health. The use of focus groups in this study enabled the study to create an in-depth insight through group interaction to generate rich information on collective perspectives and the meanings that accompany these perspectives [23].

A total of six focus groups were co-facilitated by a research team member (CR) with the support of one of three study’s public advisors (DW, VR, AP). Five focus groups were initially scheduled to take place within a familiar environment to all potential participants. The focus groups were conducted at the two Life Rooms locations at different times of the day, to facilitate flexibility for potential participants to attend. Participants were invited to attend one of the five scheduled focus groups, each focus group lasted between 60 to 90 min.

A focus group topic guide was co-developed through public involvement. This included a set of open questions that explored participants’ journey to the Life Rooms, expectations and utilised support, and their experiences of the support provided, and changes due to the service. The focus group schedule is attached in Additional file 1. Researcher (CR) and one of the public advisors (DW, VR, AP) discussed the guide prior to each focus group and agreed on a structured approach into which section they will lead on to facilitate group discussions. This included ensuring flexibility and encouraging participants open responses, this included the use of terms in open ended questions such as ‘*how has’*, ‘*in what way*’ and ‘*why*’/ ‘*Can you explain’*.

Potential participants were invited to attend one of the five scheduled focus group. Data was collected until no new, or repetitive, information emerged from the focus groups. The early process of data analysis and the identification of initial patterns indicated that there was no new emerging information. Although data saturation was achieved with five focus groups, one more focus group was conducted to ensure that no new information was emerging.

All focus groups were audio recorded and subsequently transcribed.

### Ethical consideration

Ethical approval was obtained from Mersey Care NHS Foundation Trust research committee (service evaluation study- Ref: E1/2018).

Written informed consent was obtained from all participants prior to the focus group.

### Participants and recruitment

Purposeful sampling was used in this study to identify and select individuals that have accessed and experienced the Life Rooms. Therefore, the study identified a pool of 187 Mersey Care NHS Foundation Trust service users, who had accessed services at the Life Rooms between September 2017 and April 2018. Using purposeful random sampling, all 187 Mersey Care NHS Foundation Trust service users were invited via email or letter. Potential participants who responded to the invite were contacted via telephone to answer potential questions about the study and confirm their interest in taking part. Focus group details were discussed and potential participants were able to decide if they would like to attend one of the scheduled focus groups.

### Data analysis

Focus group transcripts were analysed using a structured framework of thematic analysis [24]. Following each focus group, the Life Room’s study lead (CR) and public advisers (DW, VR, AP) shared notes captured during each focus group and discussed initial patterns that they found interesting and meaningful to the study. Continuing on this early analysis process followed each focus groups, (CR) and a Life Room staff member (VM), an academic (KB) and the public advisors (DW, VR, AP) made notes and highlighted patterns for each transcript. These initial patterns were manual clustered to form codes and potential themes, which were then combined and arranged on Nvivo11 by an academic trained in qualitative research (SH). Codes and potential themes were discussed by research group to identify main themes. They research group identified four overarching themes, these were revisited by (SH) for data validity and credibility.

### Public involvement

Three service users of the Life Rooms were involved in the study as public advisers. The public advisers were involved at all stages of the study to ensure that the public perspective was acknowledged throughout the process of this study. This involved providing contextual information and playing an instrumental role in enhancing data collection and the interpretation of the findings. Public advisers co-facilitated focus groups, which was effective in creating a relaxed environment for the conversations.

## Results

### Demographics

A total of 18 participants took part in six focus groups. The sample included participants aged 34 to 65; two thirds were female (*n* = 12), White British (*n* = 14), and single (n = 14).

The majority of participants came from disadvantaged backgrounds; 14 participants measuring 3 and below in terms of overall IMD and health-related IMD scores. 9 participants belonged to the poorest decile (IMD = 1). The IMD score was generated by an online platform (Ministry of Housing, Communities & Local Government English indices of deprivation 2019 tool) which provides an IMD score/quintile for each postcode.

### Qualitative findings

Four main themes emerged from the data: 1) social belonging: being able to just ‘be’ 2) resourceful and accessible; 3) social inclusion and connectedness; and 4) moving forward: self-development and independence.

#### Social belonging: being able to just ‘be’

Participants spoke in detail about the environment in which the Life Rooms delivers the care and support. Participants reported how they felt safe in being in such environment that facilitated easy access to support and resources. The most significant aspect was the approachability of the Life Room members (staff, volunteers and other service users) and their understanding of participants’ needs.

“*It’s just such a safe place because, even if I am not in a good mood, I get out and at least go to the Life Rooms where I know other members of staff and students and service users…everybody understands*” **FGD6_SU.**

Participants reported that the Life Rooms settings created a less clinical and non-formal environment, which facilitated individuals confidence in expressing needs and seeking support. Participants highlighted that they were able to approach the service when needed, whether it is for guidance, support, access to available opportunities or for just having an informal ‘chat’.

*“…she spoke to me about all my problems and how I was getting on. All very informal and I can cope with that, but I couldn’t cope with talking to a doctor looking at the time all the time*” **FGD5_SU.**

“*They don’t treat you like you’re a patient, they treat you like you’re just a person and I think that makes a big difference*” **FGD2_SU.**

In an environment of shared experiences and non-judgemental culture, participants reported that the Life Rooms provided a space in which people can tell their story, be honest about how they feel, and support and empower one another. This created a sense of community and belonging amongst participants. This also encouraged a sense of validation for participants.

“*If you come in here, you’re treated as a person. There’s no ‘well you’ve got that side of mental health and you’ve got that’ –you’re just treated as an individual.”* **FGD2_SU.**

#### Resourceful and accessible

Participants spoke at length about the Life Rooms setting being an all-encompassing hub, which facilitated easy access to many resources without additional financial costs, and without being subject to bureaucratic administrative process of exclusion.

*“[in other services] you would have to fill in loads of forms or you would have to apply online or you would have to get some type of funding, but since I have been here, I have done loads of stuff and I have never been asked for a penny”* **FGD3_SU.**

Participants reported that they were able to approach the Life Rooms directly for support or assistance. The ease of informal access to pastoral support reduced isolation allowing participants to self-manage better.

“*It could be is just me coming in and saying to (name) have you got time for a cup of coffee and a chat for ten minutes. That sort of conversation will sort me out if I was feeling a bit rough in the head, a bit fed up, just somebody talking to me like that, it doesn’t have to be sitting with a counsellor*” **FGD1_SU.**

On a daily basis, activities took place at the Life Rooms, and participants highlighted how there were a number of different opportunities for them to get involved, depending on their needs and interests.

*“There is always people around to talk to, you can always use the computers, you can read, and there is always craft stuff available, even if you just want to sit and chill out and do some colouring or something. I have done the dog training course, a few courses about anxiety and OCD, I took part in yoga*” **FGD4_SU.**

Choice was significant for participants. They reported having control over what they wanted to engage with and not being rushed was important in validating their ability to manage situations.

“*You can come here for say 10 minutes, 20 minutes, half an hour and just those few minutes or second or that bit of time you spend with somebody here who’s nice to you can make you feel a bit better but you are in charge of what you are doing. I think it’s really, really important and just that little bit of control can make you feel on top of the world, you can go away thinking I did something really good today*” **FGD1_SU.**

Participants indicated how they felt confident to access a learning opportunity and express how much they want to participate, i.e. whether they want to be actively involved or just sit in to listen. They also reported on how they would discuss plans to meet their individual needs with either the Life Room staff, volunteers or fellow services users. Participants felt able to trial and change plans in a way that felt appropriate for them, and still felt supported throughout this process.

“*They give you a bit of a plan and they can help you along your way and they will always support you, if you come back and you go that did not work or I am having problems with this they can support you*” **FGD5_SU.**

#### Social inclusion and connectedness

How participants framed the impact of the Life Rooms was wide ranging. However, what was discussed in-depth was how the Life Rooms enhanced social inclusion and connectedness. The Life Rooms were described as social hubs for the majority of participants. Having library facilities, group meeting spaces and a café enhanced these social interactions further.

*“I met someone there and we clicked and we had lunch in the little coffee shop there and it was like oh my god this is the first time in my adult life I have sat and had lunch with a friend”* ***FGD6_SU.***

Participants reported on how this social environment supported them feeling less isolated and lonely. They shared that the Life Rooms helped them in getting out of the house to a place where they are around others who have shared experiences. Some interactions happened instantly with other services users and, for some, these may have taken time to happen. Participants appreciated being in a place that allowed them to engage in activities on their own terms and respected their preference of wanting to be alone at times.

“*When you’re going through a mental health issues, you feel so isolated – you are the only person that this has happened to – until you come to places like this and you think, ‘Oh… I’m not’… that feeling of isolation can sort of go then.*” **FGD2_SU.**

“*I have come here a few times just to be by myself, to a safe space which is nice because obviously I can come up and get a cup of coffee if I want and sit down and they respect the fact that you want to be on your own*” **FGD5_SU.**

Relationship building is encouraged at the Life Rooms. Within courses and social groups, participants had time to interact with other individuals. They also highlighted that further interactions were encouraged by the Life Rooms staff through the introduction of service users to each other.

“*Now like I said before coming here today has allowed me to meet six other people and talk a bit whereas before I would be sat at home trying to find a 9 letter word in Countdown*” **FGD1_SU.**

Participants reported on the relationships that have been created through the Life Rooms and also indicated that relationships within their own families had improved. Since the Life Rooms is open to all, some participants reported how they had attended groups and courses with family members. Other participants reported on how the Life Rooms has given them the confidence to create new relationships. These interactions and relationships were key in enhancing participants becoming more involved and active.

“*I went through a lot a hard time about 5 or 6 years ago and I lost a lot of friends because of it and since coming here I’ve made loads more friends*” **FGD4_SU.**

#### Moving forward: self-development and independence

Another way in which participants described the Life Rooms impact was in the way it shaped their personal goals for the future. Participants describe a journey of self-development, which included self-awareness, learning to manage and look after oneself, gaining new skills, developing new hobbies and interests, enhancing confidence and gaining independence. Participants described change from not feeling the need to sit by the door when attending workshops, being able to get dressed and leave the house, and being able to complete day to day tasks independently.

“*I am out of bed, I am dressed, depending on the day whether I’m able to cope with the shower but certainly washed, dressed and today I am out the house, I am here”* ***FGD1_SU.***

*“I am able to go in and actually do my shopping instead of having to rely on people, getting my independence back is huge for me”* ***FGD4_SU.***

For many participants, self-development consisted of building skills and strategies, particularly in relation to mental health. This included developing confidence and self-esteem, or learning how to relax or manage their situation. Participants described how the different coping mechanisms that they have learnt and used gave them a sense of control and enabled them to deal with their situation.

*“I would not have been able to do it if I hadn’t have gone to Life Rooms, it give me coping mechanisms, it’s given me strategies, and its helped me to get the confidence and self-esteem because I was at rock bottom”* ***FGD1_SU.***

Participants spoke about how the Life Rooms had facilitated a process of self-exploration and learning, which for many included being able to recognise their own strengths and weaknesses and being able to face life’s challenges. This was important for many participants because it allowed them to change their behaviour in order to manage their distress more effectively, for example through understanding when they needed help or being able to communicate their needs.

*“I am now seeing things differently about myself. Since doing these courses I understand my illness more and I understand if I am having a bad day. It’s also helped me be able to voice things better as well. I can tell people more about my mental health….Even if it’s the middle of the night and I’m struggling, I know that there are people that I can phone and just say ‘I am not feeling great.’ So that’s how it’s helped”* ***FGD4_SU.***

Moving forward, participants described feeling more confident to work towards their personal goals, which included working towards employment, coming off benefits and pursuing further hobbies and interests. For many participants, being employed again was something they thought they would not be able to reach. However, participants described how the Life Rooms not only supported them in developing confidence in their own skills, but also supported them throughout the process of finding and applying for employment.

Participants also reported on how the Life Rooms provided resources that supported individuals to reconnect with the things they used to do, for example presenting, running workshops or performing.

“*I got asked if I wanted to run my own group because I spoke to a few of the people who work here about my experience… I would like to work again as you get that like self-esteem back”* ***FGD3_SU.***

## Discussion

The Life Rooms SP approach in addressing mental health needs for people living in disadvantaged areas was found to be effective by all participants. Participants highlighted key elements which they found most effective in enhancing and promoting their choice, control, and accessibility of care. Similar to other SP approaches such as the Bromley-By-Bow scheme [7], but unique in the context and its functionality. The Life Rooms model reflects a key concept of SP described in the wider literature [12, 16–18], it aims to provide a holistic approach in addressing individualised needs and consistently operating in the gaps between services. This can be at different stages, such as early on in period of distress, supporting individuals to manage their mental health, supporting those in the recovery phase where clinical services are less needed. This highlights the importance of offering and reaching individuals in ways that are accessible, and meaningful [25].

Findings highlight that the Life Room SP approach has four attributes that led to what participants described as an effective service approach of care; focus on social determinants of mental health of an individual, ease of access to the service, range of in house resources and support, and local public representation. Many SP service approaches that are currently operating in the UK, provide general wellbeing support that is not specific to but includes aspects of mental health [12]. The Life Room’s approach aims to address social determinants of mental health through the provision of community support and resources. Often, clinical primary care services are limited when the individual’s mental health problems are compounded by socio-economic factors such as financial difficulties [26]. Evidence suggests that up to 20% of GP consultations are mostly related to social issues [27]. The NHS England General Practice Forward View (2016) highlights the importance of SP in supporting structure change in the process of moving patients from and in-between professional and community settings [28].

An important step to facilitate this process is through creating early social conversation between health care services such as GPs and services users, and also community services. To enable patients moving to appropriate services that will address their needs that are influenced by socio-economic factors. This reflects the Life Rooms approach in connecting with other healthcare services to facilitate a process of accessibility for service users to appropriate community resources. For the majority of participants, being in the Life Rooms, which is a non-clinical setting, enhanced the expression of needs and the process of seeking support.

The range of Life Rooms resources did not require service users to seek support elsewhere, providing an all-inclusive engagement journey for the participants. From learning opportunities that focus on wellbeing and one-to-one support, to creative and social opportunities. This reflects one of the key objectives of the Marmot Review (2010) in addressing health inequalities by increasing access and use of quality life-long learning opportunities across social groups [29]. Participants highlighted the impact of living in a disadvantaged area and the financial burden of living costs; they reported how the Life Rooms helped in addressing some of these issues by providing free access to learning opportunities, use of community spaces, use of library facilities, and computers. This emphasises the importance of the NHS Five Year Forward View for Mental Health [14], in putting actions in place that will help improve population health and reduce health inequalities. The Life Rooms’ free access to such resources not only helped in increasing mental health literacy, which has been reported to be linked to improving mental health [25] but also, by providing such resources, activities and opportunities, this enhanced participants’ confidence and ability to create plans for the future [30]. Highlighting the importance of maximising individuals’ capabilities and control over their lives when addressing health inequalities [29].

Finally, participants reported that the Life Rooms enhanced sense of belonging. They described how the approach of the Life Rooms enabled them to be surrounded with others who have similar experiences. This meant they could come together in one place and engage in various meaningful activities, share perceptions and feel less stigmatised. This also gave them a sense of purpose by engaging in meaningful activity; an essential factor in increasing participation [10]. This was enhanced by the Life Rooms approach in involving the service users in the delivery of care, which is a key concept in contemporary partnerships for health [31]. The Life Rooms ensured that services users (public representation) are at the heart of delivery. In particular, participants reported having been given the opportunity to get involved and support new services users. Some were also encouraged and supported to run activities that they believed would benefit other users. Enhancing the main concept that SP is built in and that is partnership with voluntary services and community groups [12].

### Strengths and limitations

This study provides a unique insight into the Life Rooms SP approach in addressing social determinants of mental health. One of the strengths of this study is its methodological approach in capturing the real voices and the lived experiences of those experiencing mental health problems in social deprived areas. It captures these experiences in a group interaction between individuals who have been under mental health services and now engaging with community-based service (Life Rooms), creating collective insight and understanding to the key elements that contribute to an effective SP approach for this group.

In our view other people accessing the Life Rooms services would also find these aspects valuable. However we accept that including other people accessing the Life Rooms in the study would have helped understand the wider support and value it provides for the local community.

## Conclusion

In conclusion, the Life Rooms is an effective and unique SP model with a mental health focus to help enhance access to appropriate support and resources. It provided a range of in-house services within a local remit, involved public members as Life Room champions and ensured social inclusion by creating a space that enabled people share their lived experiences. Further research into wider benefits of this model on service users public and health services is needed. This can then support the subsequent implementation if the model on a larger scale. The implementation will be guided by an implementation plan, and thus address one of the key priorities of the NHS Long term plan 2019.

## Supplementary information

**Additional file 1.** Focus group topic guide.

## Data Availability

Qualitative data extracts are presented in the article to support the findings. The original transcripts are not available to the public as they may contain information that could compromise the confidentiality and anonymity of the participants.

## Abbreviations

ARC: Applied research collaborations
CLAHRC: Collaboration for leadership in applied health research and care
HHS: Household health survey
IMD: Index for multiple deprivation
NIHR: National institute for health research
NWC: North West Coast
SP: Social prescribing
