# Supplementary material for: Social prescribing for people with mental health needs living in disadvantaged communities: the Life Rooms model
Source: BMC Health Serv Res. 2020 Jan 6;20:19. doi: 10.1186/s12913-019-4882-7 (PMC6945402; doi:10.1186/s12913-019-4882-7)
Supplement: Supplementary file 1 — Additional file 1. Focus group topic guide. [file 12913_2019_4882_MOESM1_ESM.docx]

**Appendix 1 – Focus group topic guide**

1. **Journey to Life Rooms**

What was this? Was Mersey Care a part of this?

1. **Expectations and first impressions**

Preconceived ideas and first impressions

How does Life Rooms compare to other services that you have used? Is there a difference?

1. **Experience and impact**

What have you accessed at Life Rooms?

- Recovery College/Pathways Advisors/Peer Support/Café/Social aspect

What was that like? People/Environment/Service

Anything that was helpful or useful about Life Rooms?

- Benefits of social inclusion vs. actual services accessed at Life Rooms

Any wider impact of Life Rooms? e.g. family/relationships; life challenges; resilience

Where are you now?

- Personal growth/development/self-awareness/stigma

***Use of cards listing words from previous qualitative findings – do any of these words resonate with you?***

1. **What’s next?**

Qualitative: How might your recovery progress and move forward? Hopes, dreams and goals. Has Life Rooms led you onto further things?

1. **Feedback**

What would you change? Any improvements or suggestions?
